# Revision of the *Maddenia* clade of *Prunus* (Rosaceae)

**DOI:** 10.3897/phytokeys.11.2825

**Published:** 2012-04-17

**Authors:** Jun Wen, Wenting Shi

**Affiliations:** 1 Department of Botany, National Museum of Natural History, MRC 166, Smithsonian Institution, Washington, D.C. 20013-7012, USA

**Keywords:** *Maddenia*, *Prunus*, *Prunus gongshanensis*, revision, Rosaceae

## Abstract

The *Maddenia* clade of *Prunus* L. is monographed based on herbarium and field studies. Four species are currently accepted in this group: *Prunus himalayana* J.Wen, *Prunus hypoleuca* (Koehne) J.Wen, *Prunus hypoxantha* (Koehne) J.Wen, and *Prunus gongshanensis* J.Wen, with the last described herein as a new species. *Maddenia fujianensis* Y.T.Chang and *Maddenia incisoserrata* T.T.Yü & T.C.Ku are treated as synonyms of *Prunus hypoleuca*.

## Introduction

The *Maddenia* group has been shown recently to be nested within *Prunus* L., a genus with many economically important fruit crops and ornamental plants, such as almond and cherry blossom (Wen et al. 2008). Chin et al. (2010) showed *Maddenia* Hook.f. & Thomson as a monophyletic group closely allied with the temperate members in subgenera *Laurocerasus* and *Padus* of *Prunus*, and they transferred species of *Maddenia* to *Prunus*. The *Maddenia* clade contains a small group of trees distributed in temperate regions of the Himalaya and eastern to western China (Rehder 1940; Yü et al. 1986; Chin et al. 2010).

Hooker and Thomson (1854) described the genus *Maddenia* in honor of Major E. Madden for his contribution to the botany of the Himalayan regions. *Maddenia* was distinguished from *Prunus* based on its flowers with ten tepaloid perianth segments, i.e., petals not differentiated from sepals (Rehder 1940). The variable number of indistinguishable perianth segments was used as a diagnostic character for the approximately 40 paleotropical species of *Pygeum* Gaertn. (Backer and Bakhuizen van den Brink 1963). Nonetheless, Kalkman (1965) considered this undifferentiated perianth in *Pygeum* as an overlapping character with *Prunus*, which prompted him to transfer *Pygeum* species into *Prunus* subgenus *Laurocerasus* (Duhamel) Rehder (specifically to section *Mesopygeum* (Koehne) Kalkman).

*Maddenia himalaica* Hook.f. & Thomson was the first species described in this group. Hooker and Thomson (1854) pointed out that this plant had dimorphic flowers with 10 perianth segments and one or two pistils. The authors noted that this species of their new genus resembled *Pygeum* in its flowers and *Cerasus* in its foliage and drupe (Hooker and Thomson 1854). *Maddenia pedicellata* Hook.f. was the second described species, which differed from *Maddenia himalaica* in its much longer pedicels (Hooker 1879). Three more species, *Maddenia hypoleuca* Koehne, *Maddenia hypoxantha* Koehne and *Maddenia wilsonii* Koehne were added to the group by Koehne in 1911, while he was treating the collections by E. H. Wilson collected from central and western China. Hara (1976) published *Maddenia himalaica* var. *glabrifolia* H.Hara, which was distinguished from var. *himalaica* in its glabrous leaves except on the axis of veins. Chang (1985) described *Maddenia fujianenesis* Y.T.Chang, whichdiffered from *Maddenia hypoleuca* in *Maddenia fujianensis*’ looser raceme and its rusty tomentose inflorescence axis, pedicels and bracts. *Maddenia incisoerrata* T.T.Yü & T.C.Ku was described by Yü et al. (1985) as being similar to *Maddenia hypoxantha* and *Maddenia wilsonii* except that *Maddenia incisoserrata* had abaxially glabrous leaves with deeply serrate margins and shorter and denser racemes.

The morphological differences among the species mostly concern pubescence and color on the abaxial leaf surface, teeth on the leaf margin, and the raceme length and density (Koehne 1911; Lu et al. 2003). However, species delimitations in *Maddeina* have been controversial and identifications of the *Maddenia* group of species are often extremely difficult in the field or in the herbarium, especially concerning *Maddenia hypoleuca* and *Maddenia incisoserrata*, and *Maddenia hypoxantha* and *Maddenia wilsonii* (J. Wen, pers. observ.). Koehne (1911) emphasized leaf pubescence and Lu et al. (2003) used size of bud scales, stipule shape and bract shape to separate *Maddenia hypoxantha* and *Maddenia wilsonii*. Hara (1976) stated that *Maddenia himalaica* var. *glabrifolia* was similar to *Maddenia hypoleuca* in leaf pubescence, and suggested treating *Maddenia hypoleuca* possibly as a variety of *Maddenia himalaica*. In addition, Hara (1976) pointed out that a part of the holotype of *Maddenia pedicellata* was not *Maddenia*, but belonged to the *Cerasus* subgroup of *Prunus*. Chang (1985) used flower density of the raceme to separate *Maddenia fujianensis* from *Maddenia hypoleuca*; and Yü et al. (1985) used leaf pubescence, leaf margin, and raceme length to differentiate *Maddenia incisoserrata* from *Maddenia hypoxantha* and *Maddenia wilsonii*. Lu et al. (2003) separated *Maddenia hypolecua*, *Maddenia incisoserrata* and *Maddenia fujianensis* based on their leaf color and size, number of veins, and teeth on margin.

## Methodology

About 350 herbarium specimens from A, BM, CAS, CDB, E, GH, IBSC, K, KUN, L, MO, NY, PE and US were examined. We also conducted field studies in Fujian, Gansu, Hubei, Sichuan, Xizang and Zhejiang provinces of China. We herein provide a description of the *Maddenia* clade of *Prunus*, a key to all four species of the clade we recognized, and descriptions of each species.

## Systematics

### Description of the Maddenia clade

Trees or shrubs, deciduous. Winter buds ovoid, with several scales. Stipules caduceus, margin glandular at least on the lower part. Branchlet of first year’s growth pubescent. Leaves alternate, simple; leaf blade abaxially glabrous to tomentose; leaf margin toothed, lower part with a few to many glandular teeth, teeth simple, irregularly doubly serrate or incised-serrate. Inflorescence a terminal raceme, 8–20 flowered. Hypanthium pubescent. Perianth segments usually 10, narrowly triangular, caduceus, pubescent, not differentiated into sepals and petals. Stamens 20–45. Style slender, glabrous. Ovary 1- to rarely 2-locular, glabrous. Drupe ovoid, glabrous, dark purple to black.

Four species distributed in temperate regions of the Himalaya and eastern to western China. The morphology-based species delimitation will be tested by molecular data in our future work. At present, our available molecular data are congruent with our delimitation (Chin et al. 2010; Liu et al. in press).

### Key to species of the Maddenia clade

**Table:** 

1a	Leaf blade pubescent to villous on the abaxial surface, or at least pubescent on veins	2
2a	Leaf blade abaxially densely pubescent to rusty tomentose, lower part of margin densely with glandular teeth, bract and stipule margin glandular	*Prunus himalayana*
2b	Leaf blade abaxially pubescent or sometimes only pubescent on the veins, lower part of leaf margin only with a few glandular teeth near the base	*Prunus hypoxantha*
1b	Leaf blade glabrous on abaxial surface or with tufts of pubescence on lateral vein axils	3
3a	Leaf margin with fewer (fewer than 15) glandular teeth near the base, pubescence on abaxial vein axils usually not conspicuous, leaf base acute to rarely subcordate	*Prunus hypoleuca*
3b	Leaf margin with many (more than 20) glandular teeth at the lower 1/3 of the margin near the base, conspicuously with tufts of hairs on abaxial vein axils, leaf base subcordate to cordate	*Prunus gongshanensis*

#### 1. Prunus himalayana

J.Wen (Bot. J. Linn. Soc. 164: 243. 2010).

http://species-id.net/wiki/Prunus_himalayana

Fig. 1

##### Synonym.

*Maddenia himalaica* Hook.f. & Thomson (Hooker’s J. Bot. Kew Gard. Misc. 6: 381. 1854); non *Prunus himalaica* Kitam.

##### Type.

India. Sikkim: temperate, 8–10000 ft, bearing flowers and fruits, J. D. Hooker s.n. (lectotype: K!, here designated; isolectotype: K!).

##### Description.

**Figure 1.**
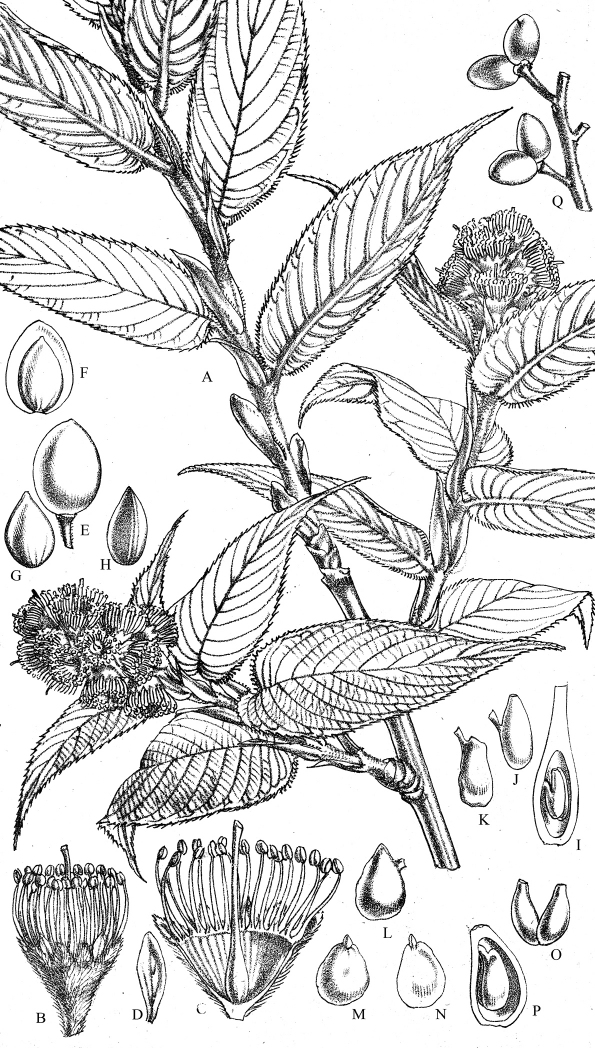
*Prunus himalayana* J.Wen **A** Habit, flowering branch **B** Flower **C** Flower laid open showing the inside of hypanthium and the gynoecium **D** Perianth segment **E** Fruit **F** Vertical section of ripe fruit **G **Front view of young fruit **H** Lateral view of young fruit **I** Ovary cut open **J,** **K** Ovules **L** Seed **M** Embryo **N** Inner face of cotyledonand plumule **O** Double ovaries **P** Vertical section of an imperfect double ovary **Q** Ripe fruits on infructescence. Figure source: from Hooker & Thomson (1854, p. 381).

Trees (2–) 3.5–10 m tall. Branches purple, slightly puberscent; branchlets of first year’s growth densely pubescent. Winter buds purplish brown, ovoid; scales to 3–23 × 3–12 mm, broadly to narrowly ovate, outside brown pubescent. Stipules lanceolate, 12–25 × 2–5.5 mm, membranaceous, caduceus, margin with glandular and fine teeth, glandular teeth at the lower 1/3–1/2, apex acuminate to acute, base rounded. Petiole 2–4.5 mm, densely brownish pubescent. Leaves ovate-oblong, elliptic to ovate, 5.5–13.5 × 2.7–6 cm, abaxially light green, densely pubescent, adaxially dull green and pubescent along the veins; margin doubly irregularly serrate to serrulate at the upper 2/3, glandularly serrulate at the lower 1/3, teeth at the margin sharp; apex acuminate, base subcordate to broadly cuneate; secondary veins 13–15 on either side of midvein. Racemes 3.5–6.5 cm, axis densely pubescent, (8–) 10–18-flowered; bracts broadly lanceolate, narrowly ovate, 6–14 × 2–5 mm, membranaceous, caduceus, pubescent on both surfaces, margin sparsely with glandular teeth. Pedicel 2.5–5 mm at anthesis, densely pubescent. Hypanthium campanulate, 3–4 × 5–7 mm, densely brownish pubescent outside, glabrous inside. Perianth segments 10, narrowly triangular, 1.5–2 × 1–1.3 mm, caduceus, pubescent. Stamens 30–40, 4–7.5 mm long; filament cream white, 3.5–7 mm; anthers oblong, pale yellow, 0.5–0.6 × 0.4–0.5 mm. Ovary glabrous, 1- or occasionally 2-locular, sometimes developing into twin fruits as shown in Fig. 1. Style slender, 5–11 mm long. Drupe ovoid, 8–10 × 5–5.5 mm, glabrous, dark purple to black.

##### Distribution.

Bhutan, Nepal, N India, N Myanmar and W China

##### Ecology.

Forest. Fl Apr-May; fr May-Aug; 2000–3500 m.

##### Specimens examined.

**Bhutan.** Wangdu Phodrung Ada, 10500 ft, 20 ft tree, 24 May 1966, S.Bower Lyon 3220 (BM). E Rudo La, tree 10–15 ft, flowers white, in rhododendron forest, 18 May 1940, Ludlow et al 18883 (BM, E). Tashiling (2100 m) – Neylong (2200) – Charikhachor (2250 m), 20 Apr 1967, fl, H.Kanai et al. 8284 (BM, E). West Donga La, 10000 ft, shrub 12–15 ft, flowers cream, calyx reddish brown, growing on edge of clearing in dense rain forest, 23 Apr 1949, fl, F.Ludlow et al. 20524 (E). Pangkar, near Lhuntse Dzong, Kuru Chu, 9000 ft, 25 Apr 1949 fl, tree 15 ft, filaments cream, anthers pale yellow, bracts and leaves dull dark red brown, F. Ludlow et al. 18756 (BM, E, PE). NE of Bhutan, Lao (Lao Chu), 9000 ft, 12 May 1949, fl, small tree, 20 ft, perianth insignificant, reddish green, stamens prominent, cream, an occasional (terminal) flower is observed with two carpels, F.Ludlow et al. 20265 (BM, PE). Tongsa,west slope below Yuto La, E of Tongsa 27°31'N, 90°34'E, margin of mossy *Tsuga*/*Rhododendron* forest, shrub 2–4 m, fr dark crimson, fleshy, 3100 m, 9 Jul 1979, A.J.C.Grierson & D.G.Long 2623 (E). West slope of Yuto La, Tongsa 27°31'N, 90°34'E, on bank in *Abies densa*/*Rhododendron* forest, shrub 1–3 m, fls green tinged crimson, 3270 m, 19 May 1979, A.J.C.Grierson & D.G.Long 1166 (E). 4.6 km NW of Pele La on the road between Wangdu Phodrang and Tongsa, mixed *Rhododendron*-coniferous forest, 3200 m, small tree ca 3 m tall, flowers white, 5 May 1984, fl, B.Bartholomew 1552 (CAS, E, PE, US). Gyelsia: 9800 ft, 27 Jun 1938, B.J.Gould 603 (K). **China.** **Sichuan:** Jingtang, 1933, T.D.Tu 4524 (IBSC). **Xizang:** S Tibet, Migyikum, Tsari Clus, 10000 ft, tree 10–15 ft, anthers brownish yellow, 23 May 1936, F.Ludlow & G.Sherriff 1672 (BM). SE Tibet, between Kumang & Nyubsang (Tsangpo Gorge), Kongbo, 9000 ft, calyx reddish brown, corolla greenish brown, filaments white, anthers brown, tree 10 ft high, growing in deciduous forest, 28 Apr 1947, Ludlow et al 13560 (E). Bomi Xian, from Bomi to Ga Wa Long alpine lake, 3453 m, 29°49.378'N, 95°42.546'E, tree ca. 7–10 m tall, 22 Jun 2009, fr, Tibet-MacArther (J. Wen et al.) 2612 (US); Bomi Xian, Tree Farm, in cut-down *Picea* forest, 3100 m, tree 3 m tall, leaves with gray lower surface, 8 Jun 1973, Qing Zang Team 73–94 (PE). Yadong Xian, A-Sang-Chun, in forest, 2840 m, tree 3–5 m, 1 Jun 1975, Qing Zang Supplement Team 750141 (PE, 4 sheets). A-Sang-Qiao, 2750 m, tree 7–8 m, fruit purplish red, 3 Jun 1975, fr, Qing Zang Supplement Team 750177 (PE). S Tibet, Trimo, Nyam Sang Chu, 11500 ft, tree 30–40 ft, perianth dark reddish green, in dense mixed forest, 23 May 1947, fl, F. Ludlow et al. 12522 (BM, E, PE). Lower Cama River, deciduous broadleaf forest, tree 7–8 m tall, leaf margin with light reddish glands, 18 Jun 1959, fr, Collector unknown 355 (PE). SE Tibet, Trulung, Po-Tsangpo Valley, Pome, tree 15–20 ft, perianth green, filaments white, anthers golden, in wet mixed forest, 3 Apr 1947, fl, Ludlow et al. 12274 (E, BM, PE). **Yunnan:** Tengchong, Houqiao, Danzha Cun, in the vicinity of Zhaobitan forest farm, ca. 26.5 direct km NW of Houqiao (Guyong), 2600 m, N facing 0–10° slope, 25°32'42.4"N, 98°13'9.4"E, subtropical evergreen broadleaf forest disturbed by agriculture and felling, shrub ca. 2 m tall, flowers green, anthers yellow, occasional, growing in forest in shade, in loam on granite, 29 May 2006, Gaoligong Shan Biodiversity Survey 30758 (CAS). **India.** **Sikkim:** Lachung East Slope, 9500 ft, 20 ft, shrub, flower purplish green, 4 May 1971, fl, S.Bower Lyon 6022 (BM). Superior, 9000 ft, May 1885, L.Pantling 46334A (K); temperate, May 1885, C.B.Clarke 46514B (K); Superior, May 1885, L.Pantling 46514C (BM); temperate, 8–10000 ft, J.D.Hooker s.n. (GH). **Nepal.** Mewa Khola: Tamur Valley, Mewa Khola, SE of Topke Gola, 9000 ft, shrub, 12 ft, 16 May 1956, J.D.A.Stainton 310 (BM). E, Nepal, Mewa Khola, 27°30'N, 87°38'E, 8000 ft, shrub 20 ft, 19 May 1974, J.D.A.Stainton 7038 (BM); E Nepal, Tamur Valley, 27°25'N, 87°35'E, 9500 ft, shrubs 15 ft, filaments white, sepals and bracts red, 26 Apr 1967, J.D.A.Stainton 5888 (BM).

#### 2. Prunus hypoxantha

(Koehne) J.Wen (Bot. J. Linn. Soc. 164: 243. 2010).

http://species-id.net/wiki/Prunus_hypoxantha

Fig. 2

##### Basionym.

*Maddenia hypoxantha* Koehne (in C. S. Sargent, Pl. Wilson. 1: 57. 1911).

##### Type.

China. Sichuan: western Sichuan, southeast of Tachien-lu, 6–9000 ft, May 1908, E.H. Wilson 909 (holotype: A!, specimen barcode 00026559; isotypes: A!, 2 sheets, E!, K!, US!).

*Maddenia wilsonii* Koehne (in C. S. Sargent, Pl. Wilson. 1: 58. 1911). non *Prunus wilsonii* (C.K.Schneid.) Koehne. Type: China. Hubei: western Hupeh, Apr 1907, E.H.Wilson 63 (lectotype: A!, specimen barcode 00026560, here designated; isolectotypes: A!, BM!, 2 sheets, E!, K!, 2 sheets, US!).

##### Description.

Shrubs to trees 1.5–10 (–15) m tall. Branches purple to dark purple, slightly pubescent; branchlets of first year’s growth densely pubescent. Winter buds purple brown, ovoid; scales to 3–20 × 3–12 mm, broadly to narrowly ovate, pubescent to slightly so on the outside surface, margin entire to slightly glandular on the upper scales. Stipules lanceolate, 12–22 × 2–6 mm, membranaceous or herbacious, caduceus, margin often with glandular teeth, especially on the lower part of the margin, apex acuminate to acute, base rounded or truncate. Petioles 2–4 mm, densely pubescent. Leaves elliptic to ovate, 4.5–14 × 2.5–6 cm, abaxially light green, pubescent or densely so especially on veins, adaxially dull green, sparsely pubescent with scattered short hairs and/or along the veins; margin mainly doubly irregularly serrate to doubly serrullate, glandularly serrulate at the lower part of the base with 2–15 glandular fine teeth; apex acute, attenuate or acuminate, base broadly cuneate to occasionally subcordate; secondary veins 16–20 on each side of midvein. Racemes 2.5–5 cm, 10–18-flowered; bracts broadly lanceolate to narrowly ovate, 5–12 × 1.5–4.5 mm, membranaceous, often caduceus, margin sparsely with glandular teeth, more or less pubescent on both surfaces. Pedicel 2–4 mm at anthesis, pubescent to densely so. Hypanthium campanulate, 3–4.5 × 3–6 mm, densely brownish pubescent outside, glabrous inside. Perianth segments 10, narrowly triangular to oblong-lanceolate, 1.8–2.4 × 0.8–1.1 mm, caduceus, densely pubescent. Stamens 25–35, 5–7.5 mm long; filaments 5–7 mm; anthers oblong, light yellow, 0.5–0.6 × 0.4–0.5 mm. Ovary glabrous, 1- or rarely 2-locular. Style slender, 7–9 mm long. Drupe ovoid, 7–10 × 4–5.5 mm, glabrous, dark purple to black.

**Figure 2.**
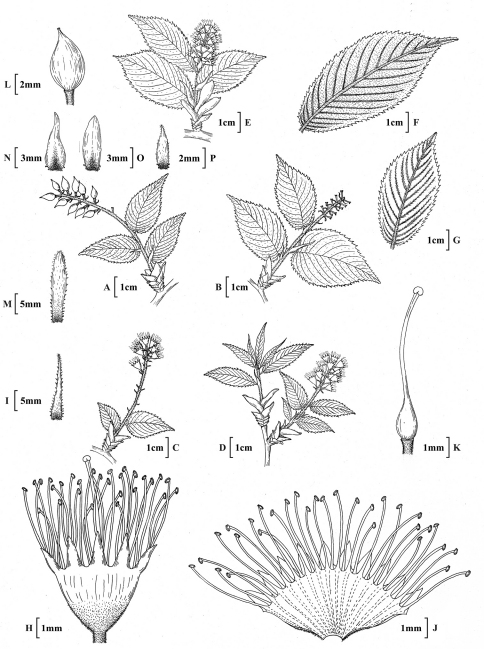
*Prunus hypoxantha* (Koehne) J.Wen**A** Habit, fruiting branch **B** Fallen fruit branch **C** End of flowering branch **D,** **E** Branch in full flower **F,** **G** Abaxial leaf surface **H** Flower **I** Stipule **J** Flower **K** Gynoecium **L** Fruit **M** Stipule **N–P** Bract **A,** **L** based on A.Henry 3759 (K) **B, F, H, J, K** E.H.Wilson 63 (E) **E, G, M, N, O** E.H.Wilson 909 (K) **D** E.H.Wilson 63 (K) & E.H.Wilson 3520 (K) **P** H.Smith 2151 (A) **C,** **I** A.Henry 3759 (A).

##### Distribution.

Western to central China.

##### Ecology.

Forests. Fl Apr-May; fr Jun-Sep; 1800–3800 m.

##### Specimens examined.

**China.** Locality & Date unknown, Collector unknown s.n. (E, speciemen barcode # E00419991). **Gansu:** Yongdeng Xian, Tulugou, Liancheng Forest Farm, Qilian Mountain, 2300 m, in clefts of rocks along stream, fruits black, 8 Jul 1991, fr, T.-N.Ho 1841 (CAS). Zhang Xian, Hedi, mountain slope, 4 Jun 1956, fr, Huanghe Team 4663 (PE). **Hubei:** Patung, Oct 1887, A.Henry 3759 (A, BM, K). **Sichuan:** Kam pars orientalis [probably in Sichuan], 1893, G.N.Potanin s.n. (A). Feb 1890, A.Henry 8952 (K); Sichuan, 26 Apr 1982, Collector unknown 41699 (CDB, 3 sheets). Ta-hsiang-ling, 2200–2500 m, 22 May 1028, H.Smith 2151 (A). Erjishan, Maliuqiao, shrub 2.5 m, fruit green, 7 Jun 1957, Forestry Team of Sichuan Agric. Colleage 5064 (CDB). Lianghokow, slope 7500 ft, tree, fruit a berry, Aug 1938, T.K.Wang & T.S.Wen 0670 (A). West Szechuen and Tibetan Frontier, chiefly near Tachienlu, 9000–13500 ft, Dec 1890, A.E.Pratt 837 (A, BM, E); Szechuen and Tibetan Frontier, chiefly near Tachienlu, 9000–13500 ft, Dec 1890, A.E.Pratt 313 (BM). Jinyangbo, Luoliangzi, 3100 m, 16 May 1959, Sichuan Economic Plant Expedition 3014 (CDB, 2 sheets). Pao-Hsin-hsien, former Mupin, Apr-Aug, 1954, T.P.Soong 38472 (IBSC); Pao-hsin-hsien, former Mupin, 1954, T.P.Soong 38226 (IBSC); Baoxing, 1933, T.T.Yü 1908 (IBSC, PE); Pao-hsin-hsien, formerly Mupin, 1954, T.P.Soong 38543 (PE); Szechuan, Mupin, May 1908, E.H.Wilson 2851 (A, E, K); Baoxing Xian, 2500 m, 29 Apr 1959, H.Y.Chuan 0237 (CDB); Baoxing Xian, 2700 m, tree, 5 m, leaves adaxially dark green, abaxially light green, fruit black, 16 Jun 1958, Collector unknown 5611 (CDB 3 sheets); Baoxing Xian, Denglonggou, 2300 m, tree 4 m, 12 Jun 1958, Collector unknown 5353 (CDB, 2 sheets); Baoxing Xian, 3100 m, leaves ovate, abaxially white and pubescent, stipule with needle-like hairs, 15 Jun 1958, Collector unknown 5404 (CDB 2 sheets). Peiping, China, Mt. Omei, Jul 1931, F.T.Wang 23489 (IBSC, 2 sheets); Emei Shi: Mt. Omei, Y.H.Tau 50455 (IBSC); Mt. Omei, 27 Jun 1940, W.P.Fang 14599 (A); Mt. Omei, 2700–3000 m, in thicket, shrub, 5–10 ft, Jul-Aug 1931, F.T.Wang 23489 (A); Mt. Omei, Y.S.Shiao 48551 (IBSC); Mt. Omei, Y.S.Shiao 48677 (IBSC); Mt. Omei, Y.S.Shiao 48712 (IBSC); Mt. Emei, Leidongping, 29°32'37.3"N, 103°19'43.3"E, 2410 m, 17 Jul 2011, fr, J. Wen 12077 (US); Mt. Emei, on the way from Jingding to Taizhiping, tree 3 m, 29°31'38.7"N, 103°19'53.7"E, 2981 m, 17 Jul 2011, fr, J. Wen 12087 (US); Mt. Emei, Taizhiping, 17 Jul 2011, J. Wen 12089 (US); Mt. Emei, Luohanpo, on the way from Chudian to Zhanglaoping, 29°34'34.6"N, 103°21'46.8"E, 1499 m, 18 Jul 2011, J. Wen 12140 (US); Mt. Omei, Leidongping, tree 4 m, 12 Aug 1957, G.H.Yang 56615 (IBSC); Mt. Omei, 2800 m, slope, tree 3 m, flower green, stinky, 9 May 1964, K.J.Guan et al. 427 (CDB, IBSC); Mt. Omei, Xixiangchi, tree 5 ft, 26 Apr 1952, J.H.Hsiung et al. 30189 (IBSC); Mt. Omei, Qixing Po, 2600 m, in forest, tree 10 m, 24 Jun 1995, H.G.Xu 2140 (MO); Mt. Omei, Tai-tzu-ning, fl white, scented, 9 Jun 1939, S. C. Sun & K. Chang 115 (A); Mt. Omei, Chiu-Lao Tung, moist shady rock, shrub, fruit black, 14 Jun 1939, S.C.Sun & K.Chang 224 (A); Mt. Omei, in thickets, shrub 3 m high, common, 10 May 1941, W.P.Fang 16540 (A); Mt. Omei, Paiyunssu, on hill slope, 28 Jul 1938, H.C.Chow 7895 (A); Mt. Omei, Taiziping, 2916 m, shrub 2 m high, flower over, 18 Jun 1942, W.P.Fang 18985 (CAS); Omei, Taiziping, 2900 m, tree 4 m, flower yellow, H.G.Xu 1993122 (MO); Omei, 26 Jun 1960, H.Y.Yi 12555 (CDB); Omei Hsien, Mt. Omei, 16 Jul 1940, T.C.Lee 2891 (US); Mt. Omei, Leidongze, 2500 m, small shrub 1.5 m, high, flowers over, 16 May 1931, W.P.Fang 18811 (A); Mt. Omei, Jun 1904, E.H.Wilson 4857 (A, BM, K); W. Szechuan, Mt. Omei, Chinting, on hill slope, 3035 m, shrub 2 m, 1938, H.C.Chow 7662 (A); Emei Shi, Emei Shan, Jingding, 25 Jun 1955, fr, China – Soviet Expedition 2356 (IBSC, PE). Hanyuan Hsien, 1400 m, 17 Apr 1930, fl, W.C.Cheng 659 (BM, IBSC, PE, 2 sheets, US); Hanyuan-hsien, 20 Apr 1930, W.C.Cheng 705 (BM, IBSC, US). Hongya Xian, 1950 m, 27 Aug 1994, Bao et al. 2524 (CDB, 2 sheets); Hongya Xian, Huangshi Gou, tree 1.5–2 m, 1770 m, 4 Jun 1994, Zi et al. 1899 (CDB, 2 sheets); Hungya Hsien, Wa-wu-shan, shrub 5 ft, 16 Jul 1931, immature fr, T.T.Yü 289 (PE). Kangding Xian, Hebinxiang, 3300 m, tree 3 ft, 2 Jun 1953, Tsiang & Hgiung 35832 (IBSC); Kangding Xian, Zheduo Shan, Zheduotang, 3160 m, tree 2 m tall, 25 Jun 1953, fr, W.P.Fang et al. 36084 (PE); Kangding Xian, 2880 m, 14 May 1981, Z.Y.Chen, 112044 (A, E); Kangding Xian, 3000 m, 8 May 1981, Z.J.Zhao 113923 (A, CDB, E, K); Kangding Xian, Kangdingqu, Sheduoshan, 2800 m, Collector unknown 15013 (CDB, 4 sheets); Kangding Xian, Zheduoshan, 3100 m, Q.S.Zhao & Z.Z.Tan 119196 (CDB); Kangding Guzan Dapingshan, 2950 m, 3 Jun 1974, Kao & Wu 111489 (CDB); Kangding Xian, Ganhaizi, 3600 m, shrub 1.5 m, 29 May 1974, B.S.Qin 06136 (CDB, 3 sheets); Kangding Xian, Kongyuqu, 3300 m, 31 May 1974, Collector unknown 05344 (CDB, 3 sheets); Kangding Xian, May 1904, E.H.Wilson 3520 (A, BM, K); Kangding, Kongyuqu, 3500 m, 2 May 1974, Chao & Wu 110869 (CDB); Kangding, 2900 m, 19 May 1974, Y.T.Wu & Q. S. Zhao 111033 (CDB); Kangding, Xianglinxiang, 3000 m, tree 3 ft, 13 Jun 1953, H.L.Tsiang 35954 (IBSC). West of Kuan Hsien, 2230 m, on slope, shrub 10 ft, branchlets brown, fruit ovate-elliptic, 15 May 1930, F.T.Wang 20842 (A, IBSC); west of Kuan Hsien, ridge of thicket, 2600 m, 16 May 1930, F.T.Wang 20882 (A, IBSC). Leibo Xian, 2200 m, by Highway 284, tree 10–15 m, leaf green, stem brown, flower light yellow, filament light green, 8 May 1983, fl, Q.-S. Zhao et al. 118401 (CDB); Leibo Xian, mountain of Gudui Shan, 2300 m, 12 May 1965, K.T.Xiang & F.Y.Wang 11521 (CDB, 2 sheets); Leibo Xian, Huangmaogeng, 19 Jun 1959, fr, 2800 m, tree 3 m, Sichuan Economic Plants Expedition 0782 (PE). Li Xian, Miyaluo, north of the town of Miyaluo on highway 213, 31°43'32"N, 100°44'39"E, 3000–3200 m, mixed deciduous broad leaved-coniferous forest with *Tilia*, *Acer*, *Prunus*, *Betula*, *Tsuga*, *Picea*, *Abies*, *Pinus* and *Larix*, along stream, small tree ca. 5 m tall, fruit black, 8 Sep 1997, Boufford et al. 27976 (A); NW Sichuan, Mao Xian & Li Xian, former Lifan Xian, 1952, T.He & Z.L.Zhou 12553 (IBSC); Li-hsien (Li-fan-hsien), tree 6 ft, branchlet green and pubescent, 10 May 1952, C.Ho & T.L.Cho 12315 (IBSC). Luding Xian, 2800 m, tree 3.5 m, G.H.Xu 25514 (CDB); Luding Xian, top of Erlang Mountain, 2900 m, 29 May 1974, fl, Sichuan Luding Team 6863 (CDB, 3 sheets); Luding Xian, 2300 m, shrub 2–3 m, 21 Apr 1984, Cao et al. 045 (CDB, 3 sheets); Luding Xian, 3800 m, in forest, 13 Sep 1980, Collector unknown 23510 (CDB 2 sheets); Luding Xian, shrub 2 m, Zhibei Team 41616 (CDB, 3 sheets); Luding Xian, Moxigongshe, 2900 m, shrub 3–4 m, 10 Jun 1980, Q.C.Wang & Z.A.Liu 22250 (CDB, 2 sheets, IBSC); Luding Xian, Hongxigongshe, 2200 m, tree 6 m, G.H.Xu 25372 (CDB, 2 sheets); Luding Xian, Yaoxigongshe, tree 2 m, 4 Aug 1982, Zhibei Team 42068 (CDB, 3 sheets); Luding Xian, Hongxi, 26 Apr 1982, Zhibei Team 41698 (CDB 4 sheets); Luding Xian, 2300 m, X.H.Hu & Q.C.Wang 22078 (CDB, 2 sheets); Luding Xian, Hongxigongshe, 2300 m, tree 5 m, 22 Apr 1981, Collector unknown 225112 (CDB, 3 sheets). Mapien Hsien, 3200 m, in thickets, small tree to shrub, 10–20 ft, bark blackish to dark brown, 26 May 1931, fl, F.T.Wang 22942 (A, PE). Meigu Xian, Huangmaogeng, 2450 m, 14 May 1983, J.Y.He & Q.S.Zhao 116772 (CDB). Mao Wen, Wolong Boshan, 2400 m, small tree 3–4 m, Dec 1963, H.C.Lee 2126 (CDB, 3 sheets). Baiyangxiang (Songpan), Baiziyawo, 1800 m, shrub 2.5–3 m, branchlet green and pubescent, abaxially pubescent on mid-vein, 7 May 1962, Yuan 0558 (CDB). Shih-mien-hsien, 1955, C.C.Hsieh 40278 (IBSC); Shih-mien-hsien, 1955, C.C.Hsieh 41335 (IBSC); Shih-mien-hsine, 1955, C.C.Hsieh 40287 (IBSC); Shih-mien-hsien, 1955, C.C.Hsieh 41145 (IBSC). Tianquan Xian, Niudingtou, 2890 m, 5 Aug 1982, Collector unknown 46017 (CDB); Tianquan Xian, Erlangshan, tree 3–5 m, 23 Jun 1951, W.P.Fang et al. 10078 (PE); Tianquan Xian, Erlangshan, 2300 m, 30 Apr 1980, Z.G.Liu & Y.B.Yang 21701 (CDB, 3 sheets, IBSC); Tianquan Xian, Erlangshan, 3300 ft, 19 May 1953, H.L.Tsiang 34169 (IBSC); Tianquan Xian, 2600 m, 5 May 1980, fl, Collector unknown 21795 (CDB, 3 sheets); Tien-chuan Hsien, 3500 m, 14 Jun 1936, K.L.Chu 2801 (E, IBSC). Xichang Xian, Luojishan, 2600 m, evergreen forest, shrub 5 m, Y.J.Li 730 (CDB, 2 sheets).

##### Cultivated plants.

**U.K. England.** Cultivated at the Royal Botanic Gardens, Kew, source from E.H.Wilson 909, May 1924, J.C.Williams s.n. (K).

##### Discussion.

Koehne (1911) described both *Maddenia hypothanxa* and *Maddenia wilsonii*. *Maddenia wilsonii* was described to be densely pubescent on the lower leaf surface whereas *Maddenia hypxantha* was pubescent only on veins. We have found a wide range of variation on the lower leaf pubescence. Furthermore, the lectotype E.H.Wilson 63 of *Maddenia wilsonii* collected from western Hubei bears very small bracts, small stipules and tomentose lower leaves. Yet the syntype E.H. Wilson 2851 (A, E, K) collected from Mupin, Sichuan has larger bracts, bigger stipules and pilose lower leaves. The holotype of *Maddenia hypoxantha* (E.H. Wilson 909) has bigger bracts, bigger stipules and hirsutulous lower leaf only on veins. Koehne (1911) indicated that the holotype of *Maddenia hypoxantha* (E.H. Wilson 909, A) was a mixed collection of *Maddenia hypoxantha* and *Maddenia wilsonii*. We examined the specimen and found that the differences between the two branches on the same sheet seem to be extremely indistinguishable. Lu et al. (2003) used size of bud scales, stipule shape, stipule appearance and bract shape, to separate the two “species”. We found that it is difficult to use these characters to consistently separate the two “species”. We herein treat *Maddenia wilsonii* as a synonym of *Prunus hypoxantha*.

#### 3. Prunus hypoleuca

(Koehne) J.Wen (Bot. J. Linn. Soc. 164: 243. 2010).

http://species-id.net/wiki/Prunus_hypoleuca

Fig. 3

##### Basionym.

*Maddenia hypoleuca* Koehne (in C. S. Sargent, Pl. Wilson. 56: 59. 1911).

##### Type.

China. Hubei: western Hubei, Hsing-Shan Hsien, bush 6–20 ft, woods, 4–6000 ft, flower greenish, May 1907, fl, E.H.Wilson 2850 (lectotype: A!, here designated, specimen barcode 00026557; isolectotypes: E!, K!, US!).

*Maddenia fujianensis* Y.T.Chang (Guihaia 5: 25. 1985). *Prunus fujianensis* (Y.T.Chang) J.Wen (Bot. J. Linn. Soc. 164: 243. 2010). Type:China. Fujian: Chong An Xian, Xing Chun, Shan Gang, Huang-Gang-Shan, in sparse forest, 1700 m, shrub 4 m, flowers yellowish green, 30 Apr 1981, fl, Wuyishan Expedition s.n.(holotype: FJSI, herbarium accession # 016600, 2 photos at PE!); synonym nov.

*Maddenia incisoserrata* T.T.Yü & T.C.Ku (Acta Phytotax. Sin. 23: 214. 1985). *Prunus incisoserrata* (T.T.Yü & T.C.Ku) J.Wen (Bot. J. Linn. Soc. 164: 244. 2010). Type: China. Sichuan: Heishui, Ma-He-Ba, 2880 m, tree 4 m tall, 16 Jul 1957, fr, X.Li 73195 (holotype: PE!); synonym nov.

##### Description.

Shrubs to trees 1.5–6 m tall. Branches dark purple, glabrous; branchlets of first year’s growth sparsely pubescent at the very young part, then glabrescent. Winter buds ovoid to narrowly or broadly so, scales 3–15 × 3–8 mm, several, imbricate, pubescent on the outer scales, but glabrous or nearly so on the inner scales, margin more or less ciliate. Stipules linear to lanceolate, 7–17 x 1–4.5 mm, membranaceous, slightly pubescent to glabrous, margin glandularly ciliate at least on the lower part. Petiole to 2–6 mm, slightly pubescent, glabrescent. Leaves ovate, elliptic to broadly so, 3.5–16 × 1.3–7.5 cm, abaxially pale green, glabrous or often pubescent in lateral vein axils, adaxially glabrous; margin doubly irregularly serrate, with 1–5 glandularly serrulate teeth at the base; apex acute, attenuate or acuminate, base acute to rounded; lateral veins 14–16 on each side of midvein. Racemes 1.5–5.5 cm, with 8–15 flowers; bracts lanceolate to narrowly triangular, 4–5 × 1–2 mm, nearly glabrous, with glandular teeth at margin. Pedicel 1.5–4 (–6) mm long, pubescent. Hypanthium campanulate, 2.5–6 × 4–7 (–9) mm, slightly pubescent to glabrescent on the outer surface, glabrous on the inner surface. Perianth segments 10, slightly unequal, narrowly triangular to lanceolate, 1.5–3 × 0.9–1.2 mm, slightly pubescent to glabrescent. Stamens 20–30, 4–7 mm long; filaments 3.5–6.5 mm; anthers oblong, 0.25–0.35 × 0.2–0.25 mm. Ovary glabrous, 1– or rarely 2–locular (see E.J.Palmer 130, CAS). Style slender, 4–8 mm long. Drupe 6–8 × 5–6 mm, glabrous, dark purple to black.

**Figure 3.**
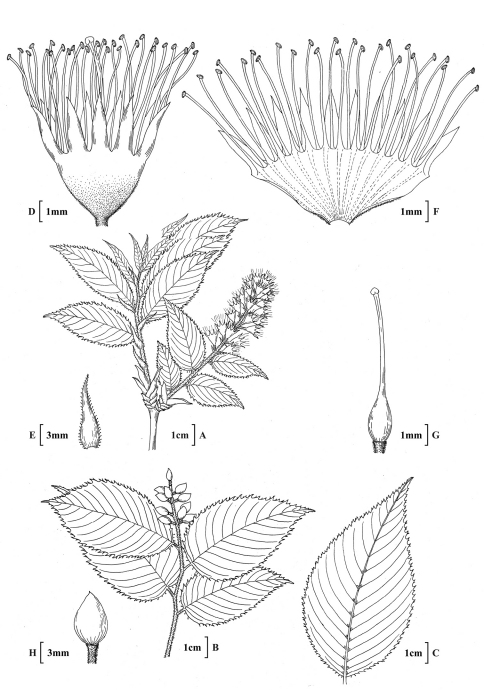
*Prunus hypoleuca* (Koehne) J.Wen **A** Habit, inflorescence branch **B** Fruiting branch **C** Abaxial leaf surface **D** Flower **E** Stipule **F** Flower **G** Gynoecium **H** Fruit. **A, D, E, F, G** based on E.H.Wilson 2850 (A) **B,** **H** J.F.Rock 12577 (A) **C** E.H.Wilson 2848 (K).

##### Distribution.

Eastern, central to western China.

##### Ecology.

Forests. Fl Apr – Jun; fr late May–Jul; 1300–3700 m.

##### Specimens examined.

**China.** W. China, Vallee de Ou-ma-hai, 2400 m, shrub, fl yellow, E.E.Maire 189/1914 (E). W. China, Avril, graud arbuste-feuil, exduques, blauehes, brousse de Ou-ma-hai, 2000 m, E.E.Maire 916/1914 (E). Jon-sian-fu, N central China, 1897, Rev. Fr. Hugh (A, BM). Huan-tou-san, N Centrial China, Jul 1899, Rev. Fr. Hugh s.n. (A, BM). **Anhui:** 29 Apr 1925, R.C.Ching 2727 (IBSC). Ping-Tien-Kan, Huangshan, Anhui, in thickets, 1700 m, shrub, 2 m, high, base of the petals purplish-red, fr green, 12 May 1979, fl & young fr, Deng & Yao 79170 (NY). Bai Ma Zhai, Tian Tang Zhai, Jinzhai, at the mountain top in the bushy woods, 1650 m, tree, fr green, 21 May 1984, K. Yao 9047 (A, CAS, K, MO, NY). S Anhui, summer 1925, fl, R.C.Ching 2727 (A, K, PE). Chongching: Nanchuan, Jinfoshan, in forest 1860 m, tree 3–5 m, flower light red, S.Y.Yi 972823 (MO). **Gansu:** Min Xian, shaddy slope, 2700 m, tree 1–1.5 m, flower light yellow, 23 May 1957, Tao He Team 3106 (MO). Pinchow District,near Kansu, W.Purdom s.n. (A). SE Kansu, 6 Apr 1919, E.Licent 4980 (BM). Tiecheliang Pass (= Lazikou Pass), hillside, east-facing, scattered scrub, 2940 m, 34°14'59"N, 103°54'59"E, deciduous shrub to 2 m, leaf mid-green and matt above, paler below, veins below noticeably raised and indented above, fruit in short racemes, green, broadly ovoid, 6–7 cm, turning red, 18 Jun 2000, Sino-British Qinghai Alpine Garden Society Expedition (SAQE) 242 (CAS; other associated material noted on the CAS specimen including DNA sample at E), herbarium specimen in fruit at HNWP, GB, and WSY). SE Kansu, Koan Kia ho et Lao Ling, 17 Apr 1919, A.E.Licent 5047 (BM, K). Qingshuishan, Menxiangdaji, 1750 m, slope, 24 Jun 1986, J.X.Yang 6838 (MO). T’ao River basin, mountains of Choni, W of Taoho, outskirts of *Picea* forest, 10000 ft, shrub 8–10 ft, May 1925, fl, J.F.Rock 12148 (A, E, K). SW Gansu, Upper Tebbu country, spruce forest among boulders, southern slopes of Minshan, 9600 ft, small tree or shrub 8–10 ft, Jun 1925, fl, J.F.Rock 12531 (A, US). Hsia Mo K’ou, near Lichen, 2000–2300 m, shrub up to 13 ft, woods, 8 Jul 1923, R.C.Ching 391 (A 2 sheets, E, NY, US). SW Kansu, T’ao River basin, in forest among spruces, Choni, 9000–10000 ft, small tree, 15 ft, Jul 1925, J.F.Rock 12577 (A, E, K). Baiyanglin, Huanghe Expedition 395 (PE). Baiyanglin, Huanghe Expedition 408 (PE). Min Xian, Wutaishan, on the top of mountain, moist area, slope, shrubby area, 2400 m, shrub 2 m tall, 26 May 1957, fl, Huanghe Expedition 3241 (PE). Yuzhong, 2450 m, in sparst forest, tree, 30 May 1983, Z.Y.Zhang 19178 (MO); Yuzhong Xian, Xinglongshan, 2600 m, 9 Jun 1990, X.Pu 558 (MO). Tulugou, Yongdeng Xian, in shrub forest, 2500 m, 10 Jul 1990, G.H.Wang 886016 (MO); Gansu,Lianhuashan, Kangle Xian, in shrub forest, 2700 m, 21 Jun 1991, G.H.Wang 91056 (MO). **Guizhou:** Zhengyi Shi, Shanpeng Dist., Xianrenshan, roadside, in sparse forest, 1950 m, tree 3–4 m tall, sepals green with reddish tint, 9 Apr 1959, fl, North Guizhou Team 0086 (PE, 3 sheets). Henan: Lushi Xian, Dayandi, on the way to Yuhuangfu, in dense forest, valley, 1760 m, 10 Jul 1959, fruits black, L.-Z. Chen & S-H.Dong 34526 (PE). **Hubei:** Changyang, 4 Apr 1900, E.H.Wilson 429 (A, E). Patung, W. China, 26 Apr 1900, E.H.Wilson 429 (US). Western Hubei, Jul 1907, fr, E.H.Wilson 2848 (A, BM, E, K), May 1907, fl, E.H.Wilson 2849 (A, BM, E, K). Shennongjia, Laojunshan, 2150 m, in dense forest, fruits black, 9 Jul 1976, fr, Hubei Shennongjia Expedition 31020 (PE); Shennongjia, Yanziya, 2000 m, slope, tree 4 m, Jun 1986. S.-H.Yang 18 (IBSC). Laojunshan, near Medicinal Herb Garden, in dense forest, tree 5–7 m tall, 31 May 1957, fr, Y.Liu 00626 (PE, 3 sheets). Xingshan Xian, Laojunshan, in dense forest, tree 6–8 m, fruits purple, 27 May 1957, fr, Y.Liu 496 (PE, 3 sheets); Xingshan, in dense forest, 1400–1450 m, tree 5–7 m, 31 May 1957, fr, H.J.Li 2298 (PE); Xingshan Xian, 1600 m, in dense forest, tree 6–8 m, fruit reddish purple, 27 May 1957, L.Ying 496 (IBSC, 2 sheets). **Jiangsu:** C.W.Yao 2748 (IBSC). **Jiangxi:** Qianshan Xian, Huanggangshan, in dwarf montane forest, 27°51.605'N, 117°47.003'E, 2070 m, 15 Jul 2011, juvenile plant about 1 m tall, J. Wen 12069 (US); Qianshan Xian, Huanggangshan, in dwarf montane forest, 27°51.605'N, 117°47.003'E, 2070 m, 15 Jul 2011, tree ca. 3.5 m tall, growing in rock crevice, fruit blackish purple, J. Wen 12071 (US); Qianshan Xian, Wuyishan, Huang-gang-shan, 2070 m, shrubs 1.5 m tall, old branches purple, new branches green, 7 May 1984, fl, Z.X.Yu 840010 (PE). **Shaanxi:** Huayin Xian, Huayang Commute, 1300 m, tree 3–4 m, petal deciduous, 24 Apr 1978, Zh.-Y.Zhang & Ch.-Sh.Liu 17587 (IBSC). Ningxia Xian, Juyangbei, 1360 m, small tree 1360 m, flower yellowish green, 24 Apr 1993, G.H.Tian & L.Tian T934014 (MO). Baoji, Weibin District, 1700 m, in slope forest, tree 4 m, leaves adaxially green abaxially light green, young fruit purplish green, 26 May 1977, Z.X.Hu & Y.H.Guo 210 (IBSC). Taibaishan, slope, 2850 m, 1 Jun 1965, C.L.Tang 1447 (IBSC). Feng Xian, Zhoujiazhuang, slope forest, 1530 m, fruit black, 2 Aug 1996, Y.S.Lian et al. 96181 (MO). Yang Xian, Huayang, Daping, forests, 2400–2700 m, 4 Jun 1999, G.H.Zhu et al. 1748 (MO). Ningxia Xian, Caiziping, 1850 m, tree 3 m, 20 Jul 1990, P.H.Yang 90336 (MO); Ningxia Xian, Caiziping, 1600 m, tree 2.5 m, 21 Jul 1990, P.H.Yang 90390 (MO). Western Shen-si, Lungchow, Kuan Shan, 2000 m, 3 Jul 1922, Native collectors 2352 (A). Tai-pei Shan, fruit black, 8000 ft, 8 Jul 1910, W.Purdom 436 (CAS, E, K, US). Taipaishan, near Haopingszu, 1500 m, in valley, tree with unpleasant odor, to 5 m, bark chestnut brown, 18 Apr 1937, fl, T.P.Wang 6526 (PE). Taipaishan, near Haopingszu, 1500 m, in valley, tree with unpleasant odor, to 5 m, bark chestnut brown, 18 Apr 1937, fl, T.P.Wang 6551 (PE). **Sichuan:** W. China, 2400 m, Su-tchuen oriental, Tchen-Kéou-Tin, R.P.Farges s.n. (K, H2010101913). Lian Ying Zhai, Baiguo forest Farm, Wuxi Co., damp valley, 1480–1630 m, shrub 3–5 m, gruit green, 30 May 1996, C.Z.Gu 960732 (MO). Tchen-Kéou-Tin, R.P.Farges s.n. (E, E00419987). Maowenfengyi Keyaozhai, Zhongshan, SW shaddy slope, 2200–2500 m, tree 2–3 m, stem black brown, branchlet light brown, abaxially light green, vein conspicuous, pubescent at vein axis, adaxial leaf dark green, fr purplish black, 21 Jun 1959, Mao Wen Team 2832 (CDB 2 sheets). Wanyuan Xian, Hua’E’Shan, top of the mountain, 2200 m, small tree 2–2.5 m, stem green, branchlet yellow and pubescent, leaf adaxially dark green and abaxially whitish green, fruit green, B.L.Li 2035 (CDB). Sichuan, Nanjiang Xian, Zhongshanqu, shaddy slope, 1600 m, tree 2 m, stem purplish red, leaves simple and alternate, pubescent, abaxially whitish, adaxially green, fruit small, reddish green, 9 Jun 1959, B.W.Zuo 2850 (CDB). Sichuan, Heishui, Ma-He-Ba, 2880 m, 16 Jul 1957, fr, tree 4 m tall, X.Li 73195 (CDB); Heishui, Shidiaolu Xiang, Kuguazhai, mountain slope, 2900 m, uncommon, shrub 2–4 m tall, 28 May 1959, fl, Sichuan Economic Plant Expedition 1251 (CDB, 3 sheets, PE, 2 sheets). Sichuan, Donqrergo, in silva mixta primcera, ca. 3700 m, 8 Aug 1922, H.Smith 3499 (A, MO). Pingwu Xian, H. L. Tsiang 10054 (PE). Wushan Xian, Chaoyang Ping, Wenjia Chao, 2000 m, in forest at riverside, tree 2 m, 4 May 1958, fl, G.H.Yang 57953 (PE); Chaoyang Ping, mountain slope, 2100 m, shrub 2 m tall, 6 May 1958, fl, G.H.Yang 57979 (PE, 2 sheets). Nizhi Ping, 1800 m, streamside, tree 3 m tall, young leaves purple, sepals 5, green, with reddish tint, triangular, petals 5, lanceolate, smaller than sepals, 13 Apr 1958, fl, G.H.Yang 57684 (PE, 2 sheets). Sichuan, Pingwu, Xutang Commune, tree 1.5 m, mountain top, shady slope, Dec 1961, X.N.Tang 42 (CDB 2 sheets). Wushan Xian, Liziping, 1800 m, tree 3 m, young leaves purplish red, 13 Apr 1858, G.-H.Yang 57684 (IBSC). **Zhejiang:** Anji Xian, Baofu Township, Tianmushan area, 30°23.976'N, 119°26.441'E, 1336 m, tree 4–6 m tall, in wet area, 28 Apr 2010, fl, J.Wen 11291 (US, 2 sheets); Anji Xian, Longwangshan, 950 m, small tree 4.5 m, perianth segment green, anther yellow, petal absent, 30 Mar 1997, L.P.Yu & M.B.Deng 97099 B (MO).

##### Cultivated plants.

**U.K. England:** Cultivated at the Royal Botanic Gardens, Kew, Arboretum South, 19 March 1969, Kew Accession Number 47–61 (K); cultivated at the Royal Botanic Gardens, Kew, England, Arboretum South, 22 Apr 1969, Kew Accession Number 47–61 (K). **U.S.A.** Seed from E. H. Wilson material, originally from Suungpan, W Sichuan, 1910, cultivated in the Arnold Arboretum, #6120, 21 Apr 1938, E.J.Palmer 130 (CAS); cultivated in Arnold Arboretum, #6120, Bussey Hill, originally from W. China, E.H.Wilson 4008, 21 Apr 1931, Kobuski & Roush s.n. (K). Seed from Wilson 4008, Sungpan, W. Sichuan, China, 1910, cultivated in the Arnold Arboretum #6120, 25 Apr 1941, R.B.Clark 126 (MO).

##### Discussion.

Yü et al. (1985) stated that *Prunus incisoserrata* was similar to *Prunus hypoxantha* and *Maddenia wilsonii* except that the leaves of *Prunus incisoserrata* are abaxially glabrous; the margin is deeply serrated; and inflorescence is shorter and denser. The character of glabrous abaxial leaf blade is similar to that of *Prunus hypoleuca*. We also observed variations in the depth of the leaf teeth and inflorescence length of specimens of *Maddenia incisoserrata* and *Prunus hypoleuca*. In fact the type specimen of *Prunus hypoleuca* bears leaves with deeply serrated teeth at the margin. Chang (1985) compared *Maddenia fujianensis* with *Prunus hypoleuca*. *Prunus hypoleuca* was said to have a pubescent and dense inflorescence. Nonetheless, the inflorescence of *Maddenia fujianensis* is also pubescent and dense. The differentiating characters among *Prunus hypoleuca*, *Maddenia incisoserrata* and *Maddenia fujianensis* seem continuous. We thus treated *Maddenia incisoserrata* and *Maddenia fujianensis* as synonyms of *Prunus hypoleuca*, which has the nomenclatural priority.

#### 4. Prunus gongshanensis

J.Wen sp. nov.

urn:lsid:ipni.org:names:77118671-1

http://species-id.net/wiki/Prunus_gongshanensis

Fig. 4

##### Type.

China. Yunnan: Gongshan Xian, Gongshan, on the way from Qingnatong to Anwalong, 3100 m, small tree 4 m tall, in the valley in shrublands, 31 May 1979, fl, flowers white, common, Lujiang Expedition 790292 (holotype: KUN!; isotype: KUN!).

*Maddenia himalaica* var. *glabrifolia* H.Hara, J. Jap. Bot. 51(1): 8. 1976. Type: Bhutan. Rukubi: Chendebi, 2600 m, 14 Apr 1967, H. Kanai, G. Murata, H. Ohashi, O. Tanaka & T. Yamazaki 4191 (holotype: TI; isotype: E!).

##### Description.

Small trees 4–8 m tall. Branches purple, shiny glabrous; branchlets of first year’s growth pubescent. Winter buds purplish brown, ovoid; scales to 4–20 × 3–15 mm, ovate, outside brown pubescent but glabrescent. Stipules lanceolate to broadly so, 12–25 × 2–8 mm, membranaceous, margin with glandular teeth, apex acuminate to acute. Petiole 2.5–5 mm, brownish pubescent. Leaves oblong, elliptic to ovate, 5–13 × 2–6 cm, abaxially light green, nearly glabrous, only pubescent on lateral vein axils, adaxially dull green and glabrous, margin doubly irregularly serrate at the upper 2/3, glandularly serrulate at the lower 1/3, teeth at the margin sharp, apex acuminate, base subcordate to broadly cuneate; secondary veins 20–24 on either side of midvein. Racemes 4–6 cm, brown pubescent, 12–16 flowered; bracts lanceolate to ovate, 4.5–6 × 2.5–3.4 mm, membranaceous, margin entire to serrate. Pedicel 2.5–4 mm at anthesis, densely brown-pubescent. Hypanthium campanulate, 4–6 × 4–9 mm, brownish pubescent outside, glabrous inside. Perianth segments 10, narrowly triangular to lanceolate, 1.5–3.2 × 0.7–1 mm, caduceus, pubescent. Stamens 25–45, 5.5–7 mm long; filaments 5–7 mm; anthers oblong, 0.3–0.35 × 0.25–0.3 mm. Ovary glabrous, 1-locular. Style slender, 7–9 mm long. Drupe ovoid, glabrous, 8–9 × 5–6.5 mm, dark purple to black.

**Figure 4.**
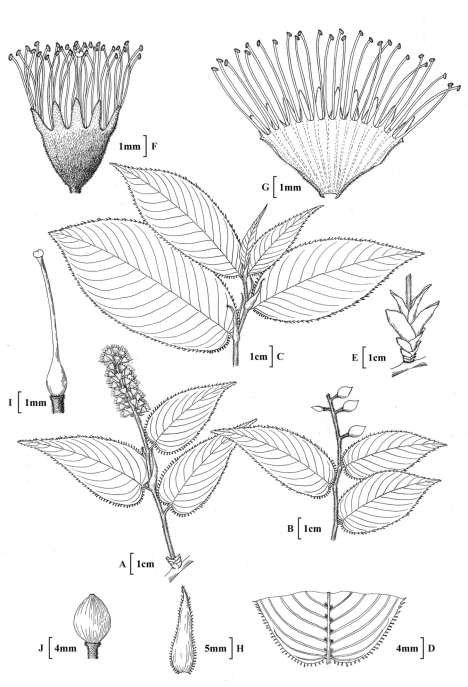
*Prunus gongshanensis* J.Wen**A** Habit, inflorescence branch **B** Fruit **C** Leaf branch **D** Abaxial leaf surface **E** Bud scale **F,** **G** Flower **H** Stipule **I** Gynoecium **J** Fruit **A, C, D, F, G, H, I** based on Gaoligong Shan Biodiversity Survey 20474 (CAS) **E** Gaoligong Shan Biodiversity Survey 20059 (CAS) **B,** **J** Nan Shui Bei Diao Team 8992 (KUN).

##### Distribution.

Western China, Bhutan, Myanmar, Nepal, and northern India.

##### Ecology.

Shady valleys and forests. Fl. Mar–Jul; fr. Jun–Jul; 2100–3500 m.

##### Etymology.

This species is named after the mountain range, the Gaoligong mountains or known as Gongshan, where this species was first recognized.

##### Specimens examined.

**Bhutan.** Griffith 2057 (GH, K); Gasa (2600) – Pari La (3550) – Chamsa (3500), 14 May 1967, Kanai et al.12806 (E). Gasa (2600 m) – Pari La (3550) – Chamsa (3500 m), 14 May 1967, Kanai et al.12895 (BM). Buhtan, Mishina (1300 m) – Dochu La (3950 m) – Thimphu (2250 m), 28 Apr 1967, Hara et al. 10319 (BM); Punakha district,forest slopes between Dochong La and Menchunang 27°30', 89°45', evergreen oak forest, large shrubs or small tree 8 m, deciduous young leaves dark red, stamens creamy, sepals and petals crimson, 2750 m, 19 Apr 1982, A.J.C.Grierson & D.G.Long 4481 (E, K). **China. Yunnan:** Fugong Xian, Yaping Xiang. Between the Shibali logging station and Yaping pass,ca. 8.5 km W of Shibali, on the road from the Nujiang to Yaping pass, E side of Gaoligong Shan, 3106 m, 27°11'6"N, 98°43'12"E, moist forests with thickets, tree, 6–7 m tall, fl white, growing in forest, 8 May 2004, Gaoligong Shan Biodiversity Survey 20474 (CAS). Fugong Xian, Yaping Xiang, in the vicinity of Yaping near the Myanmar border, E side of Gaoligong Shan, 3500 m, 27°12'37"N, 98°42'33"E, *Rhododendron*-bamboo thicket, tree, 5 m tall, in moist area, along road, evergreen forest. 2 May 2004, Gaoligong Shan Biodiversity Survey 20059 (CAS). Gongshan Xian, Cikai Zheng, E side of Gaoligongshan, W of Gongshan, along the Pula He on the trail from Qiqi to Dongshao Fang and the Dulong Jiang Valley, 2770–3050 m, 27°42'28"N, 98°29'49"E, conifer-deciduous forest with mostly conifers at the upper elevation, deciduous tree ca. 7 m tall, young fruit green, growing along trail in sun, 15 July 2000, H.Li 12626 (CAS); Gongshan Xian, Dulongjiang, Dongshanpian, mixed forest, alt 2100m tree 8m tall, leaf purplish green, flower raceme, fruit green, 15 Apr 1991, Dulongjiang Expedition 5876 (CAS); Gongshan Xian, Cikai Zheng, E side of Gaoligongshan, W of Gongshan, along the Pula He on the trail from No. 12 bridge to Dongshaofang and Dulong Jiang Valley, 3000 m, 27°41'42.5"N, 98°29'5.8"E, primary evergreen broad-leaved forest, growing on the roadside, tree ca. 2 m tall, bud green, 1 May 2002, H.Li 14796 (CAS); Gongshan Xian, Mt. Kenicunpo, eastern and western slopes, Salwin and Irrawady divide, tree 10–12 ft tall, flowers yellow, middle slopes in forest, 10000 ft, May-July 1932, J.F.Rock 22026 (A, BM, E, K, NY); Gongshan Drungzu Nuza Zizhixian, Cikai Zheng. E side of Gaoligong Shan, W of Gongshan, along the Pula He on the trail from No. 12 bridge to Dongshaofang and Dulong Jiang valley, 3000 m, 27°42'54"N, 98°30'8"E, primary evergreen broad-leaved forest, tree ca. 6 m tall, calyx green, stamens light yellow, growing on the roadside, 1 May 2002, H.Li 14808 (CAS); Gongshan Xian, Dulongjiang, Dizhenggang, Dongshanan mixed forest, 2100 m, deciduous tree 8 m tall, 15 Apr 1991, fl, Dulongjiang Expedition 5876 (KUN); Gongshan, at the divide of Chang River and Lu River, Doyon – Lumba, 3000–3200 m, frequent, tree, 25 Aug 1940, sterile, K.M.Feng 6922 (KUN); Gongshan, Bingzhongle, Songta, slope, in *Tsuga* forest, 2900 m, tree, 25 Jun 1982, late fl & young fr, Qing Zang Team 7537 (KUN, PE, 3 sheets); Gongshan, Songta Snow Mountain, 3200 m, 17 Jun 1960, fr, Nan Shui Bei Diao Team 8992 (KUN, PE); on the way from Gongshan to Dulong, Jidu to Dongshaofang, in *Tsuga* forest, 2800 m, tree 5–8 m tall, 22 Jul 1982, fr, lower leaf surface grayish green, fruits green, turning red, Qing Zang Team 8425 (PE, 3 sheets). Weixi Xian, Anyi, Shimian Chang, Ershui Tang, 3170 m, tree, 7 m, 3 May 1960, slope, in *Betula* forest, Nan Shui Bai Diao Team 8421 (KUN). **Xizang:** Tibet, Burma-Tibet Frontier, flowers white, practically over, a small soft wooded tree in thickets, rare, leaves glabrous, except petiole which is pubescent, teeth at the base of leaf with glandular hairs, inflorescence & shoots also pubescent, stamens indefinite, style 1 simple, ovary 1-celled, 1-seeded, first noticed, just in flower, on 12 March and not seen again, 1950, F.Kingdon-Ward 9340 (E); Tsarong, SE Tibet, in thickets by streams in side valleys on the Salwin-Kiu Chiang divide, N.W. of Si-chi-to, 28°24'N, long. 98°24'E, 10000 ft. May 1922, fl, G.Forrest 21598 (A, BM, E, US); Tsarong, SE Tibet, foliage only, Oct 1922, G.Forrest 22836 (A, E). Medog Xian, Lage to Hanmi, tree 8 m tall, 3000 m, in *Abies* forest, fruit purple, 26 Jun 1980, fr, W.-N.Chen 10630 (PE); Lage to Hanmi, 2800–2400 m, in *Tsuga* forest, 26 Jun 1980, fr, W.-N.Chen 10616 (PE, 3 sheets). Tibet, Rong To Valley, 6000–9000 ft, flower white, appearing with the leaves, a shrub, abundant in the temperate forest, particularly in damp places at lower levels, 25 May 1933, fl, F.Kingdon-Ward 10366 (BM, PE). **India.** **Assam:** Manda La, Balipara frontier, 9000 ft, in the drier forest, a small tree with white flowers, 2 Aug 1933, F.Kingdon-Ward 11471 (BM). **Sikkim:** S Dentam, 27°12'N, 88°8'E, 9500 ft, filaments white, calyx reddish, 25 Apr 1966, J.D.A.Stainton 5358 (BM). **Myanmar.** Tibet-Burma frontier, northern Myanmar, Advance Base, Seinghku Wang, 10000 ft, flower white, very fragrant, small shrub in thickets or in the open steef faces, 1 Jun 1926, F.Kingdon-Ward 6811 (K). **Nepal.** Ilam, NW Ilam, 9000 ft, tree 20 ft, 4 May 1981, J.D.A.Stainton 8264 (BM).

##### Discussion.

*Prunus gongshanensis* is similar to *Prunus hypoleuca* in its glabrous leaf abaxial surface and the pubescent axils on vein joints. It also resembles *Prunus himalayana* in the many glandular teeth at the lower part of leaf margin (c 1/3 of the leaf base). *Prunus gongshanensis’* relatively large bud scales, stipules and bracts are similar to those of *Prunus hypoxantha*. *Prunus gongshanensis* seems to share a close relationship with the other three species. However, *Prunus gongshanensis* stands out from the rest of the group by its subcordate to cordate leaf bases. It differs from *Prunus himalayana* and *Prunus hypoxantha* in its almost glabrous lower leaf surfaces (except on vein axils). It is also distinguishable from *Prunus hypoxantha* and *Prunus hypoleuca* by its highly glandular lower part of the leaf margin. Furthermore, the leaves of *Prunus gongshanensis* are much more mature than those of *Prunus hypoleuca* at anthesis.

### Excluded name

#### Maddenia pedicellata

Hook.f. (Fl. Brit. India 2: 318. 1878).

##### Type:

India. Mishmi Hills, Griffith s.n. (holotype: K!, K000396854).

##### Note:

As noted by Hooker (1878),*Maddenia pedicellata* was described based on a fragmentary specimen collected by Griffith from the Mishmi Hills. It was stated to be characterized by subcorymbose flowers on long slender pedicels on a short peduncle in unripe fruits (Hooker 1878, p. 318). The type was mounted on a sheet with *Prunus gongshanensis* collected from Bhutan (Griffith 2057, K) in flower condition, although Hara (1976) said it was a collection of *Maddenia himalaica*, which should bear highly pubescent leaves. This specimen has glabrous lower leaf surfaces with only traces of hairs at the axils of veins on the lower leaves. Our examination of the type material confirms Hara’s treatment of *Maddenia pedicellata* as a synonym of *Prunus cerasoides* Buchanan-Hamilton ex D. Don (Hara 1976; also see Lu et al. 2003).

## Supplementary Material

XML Treatment for Prunus himalayana

XML Treatment for Prunus hypoxantha

XML Treatment for Prunus hypoleuca

XML Treatment for Prunus gongshanensis

XML Treatment for Maddenia pedicellata
